# A report on radioactivity measurements of fish samples from the West Coast of Canada

**DOI:** 10.1093/rpd/ncu150

**Published:** 2014-05-02

**Authors:** Jing Chen, Michael W. Cooke, Jean-Francois Mercier, Brian Ahier, Marc Trudel, Greg Workman, Malcolm Wyeth, Robin Brown

**Affiliations:** 1 Radiation Protection Bureau, Health Canada, 775 Brookfield Road, Ottawa, CanadaK1A 1C1; 2 Pacific Biological Station, Fisheries and Oceans Canada, 3190 Hammond Bay Road, Nanaimo, CanadaV9 T 6N7; 3 Department of Biology, University of Victoria, P.O. Box 3020, Station CSC, Victoria, CanadaV8W 3N5; 4 Institute of Ocean Sciences, Fisheries and Oceans Canada, P.O. Box 6000, Sidney, CanadaV8L 4B2

## Abstract

Even though many studies have shown that radioactive caesium levels in fish caught outside of Japan were below experimental detection limits of a few Bq kg^−1^, significant public concern has been expressed about the safety of consuming seafood from the Pacific Ocean following the Fukushima-Daiichi nuclear accident. To address the public concerns, samples of commonly consumed salmon and groundfish harvested from the Canadian west coast in 2013 were analysed for radioactive caesium. None of the fish samples analysed in this study contained any detectable levels of ^134^Cs and ^137^Cs under given experimental setting with the average detection limit of ∼2 Bq kg^−1^. Using a conservative worst-case scenario where all fish samples would contain ^137^Cs exactly at the detection limit level and ^134^Cs at half of the detection limit level (to account for much shorter half-life of ^134^Cs), the resulting radiation dose for people from consumption of this fish would be a very small fraction of the annual dose from exposure to natural background radiation in Canada. Therefore, fish, such as salmon and groundfish, from the Canadian west coast are of no radiological health concern.

## INTRODUCTION

Releases of radioactive contaminants, especially the long-lived radioactive caesium-137 (^137^Cs), into the Pacific Ocean following the Fukushima-Daiichi nuclear accident in 2011 have raised public concerns about seafood safety. The contaminated ocean water plume propagates eastward towards the west coast of North America^(1, 2)^. There were simulations projecting that the contaminated water plume will reach the northwestern American coast by early 2014, and ^137^Cs in shelf waters north of 45°N will reach a peak concentration of a few tens of Bq kg^−1 (1)^. It is well known that many fish species can migrate long distances in the ocean. Therefore, public concerns about the safety of consuming seafood have extended to seafood caught off the west coast of Canada far away from the east coast of Japan.

Radiation surveillance in Canada has been significantly enhanced since the 1986 Chernobyl Nuclear Power Plant accident by virtue of Canada's participation in, and access to the International Monitoring System of the Comprehensive Nuclear-Test-Ban Treaty Organisation (CTBTO)^(3)^, the expansion of Health Canada's Canadian Radiological Monitoring Network (CRMN)^(4)^ monitoring stations and the creation of a nationwide real-time Fixed Point Surveillance Network (FPSN)^(5)^, and the integration of Environment Canada's atmospheric transport modelling capability into Health Canada's operation. Environmental surveillance activities from the 26 monitoring stations of the CRMN, the 76 monitoring stations of the FPSN and the 80 globally distributed monitoring stations of the CTBTO (including 4 situated in Canada) provide sensitive and comprehensive coverage for radionuclide detection and impact assessment in Canada. Notably in the case of the Fukushima-Daiichi nuclear accident the FPSN provided *in situ* determination that there was no perceptible change in measured Canadian background radiation levels (total air kerma doses) despite the detection of spikes in concentrations of some anthropogenic radionuclides originating from the Fukushima accident.

A small amount of airborne radioactivity was consistently detected in Canada for ∼1 week after the Fukushima-Daiichi nuclear power plant accident and remained detectable for another week before becoming too small to be measured. The associated radiation dose during this period was indistinguishable from the background radiation dose attributable to natural sources measured by Health Canada^(6)^.

Fisheries and Oceans Canada established a monitoring programme to detect the arrival of Fukushima radioactivity in the water columns of the eastern North Pacific and Arctic Ocean^(7)^. While trace amounts of ^134^Cs and ^137^Cs have been detected, the measured radioactivity levels were very low, well below historical levels resulting from the atmospheric nuclear weapons testing in the 1950s and 1960s, and orders of magnitude less than Health Canada's Guidelines for Drinking Water^(8)^. Despite recent reports about ongoing leaks from the Fukushima-Daiichi site, the levels of environmental radiation measured in Canada are consistently within the range of radiation levels normally detected, and therefore are not a public health concern for Canadians.

Nonetheless, significant public concern has been expressed about the safety of consuming seafood from the Pacific Ocean, specifically with regard to fish caught off the west coast. The Canadian Highly Migratory Species Foundation tested samples of Northern Pacific Albacore Tuna, a highly migratory species that spend their lives roaming the North Pacific Ocean, obtained from 2010 (from storage, pre-Fukushima), 2011, 2012 and 2013 for radioactive contamination^(9)^. For all samples tested, no radionuclides of man-made origin were detected at or above the experimental detection limits.

Based on the Japanese Fisheries Agency's monitoring data reported in July 2013^(10, 11)^, radioactive caesium concentrations in fish products from Fukushima and adjacent prefectures in Japan were evaluated^(12)^. The study provided the upper boundary dose, and subsequent risk estimates if individuals only consume fish caught from Fukushima and adjacent prefectures in Japan. Even in this extreme circumstance, there is no radiological health concern.

Many other studies have shown that radioactive caesium levels in fish caught outside of Japan were below the minimum detectable concentration (MDC) of ∼2 Bq kg^−1^. These findings are summarised in the document, Preliminary Dose Estimation from the Nuclear Accident after the 2011 Great East Japan Earthquake and Tsunami prepared by the World Health Organisation^(13)^. To complement these findings from a Canadian perspective, more than 60 fish samples harvested from the Canadian west coast by Fisheries and Oceans Canada in 2013 were provided to Health Canada for radiological analysis. The results of these radioactivity measurements are reported here.

## FISH SAMPLES AND RADIOACTIVITY ANALYSIS

Fish samples harvested from the Canadian west coast by Fisheries and Oceans Canada included adult salmon and groundfish. Adult Coho Salmon (*Oncorhynchus kisutch*) and Chum Salmon (*Oncorhynchus keta*) were obtained from two hatcheries located on the east coast of Vancouver Island, British Columbia (Puntledge Hatchery and Big Qualicum Hatchery). Both species spawn in freshwater late in the fall. Their eggs incubate in the gravel over winter. Once the eggs hatch, the fry remain in the gravel for a few weeks. Chum Salmon fry will then migrate to sea and rear in the near-shore environment for several weeks. They then migrate northward along the continental shelf and rear in the Northeast Pacific Ocean for 3–5 y prior to returning to their natal rivers to spawn. Coho Salmon fry generally spend a full year in freshwater prior to initiating their downstream migration to sea. They remain within a few hundred kilometres of their natal river where they feed on a mixture of zooplankton and fish. Migration to open waters is highly variable for Coho Salmon, with individuals remaining their entire life on the shelf and others rearing in the Northeast Pacific Ocean, even within a single population. Coho Salmon only spend 18 months in the ocean, and as a consequence, do not migrate as far west in the Northeast Pacific Ocean as Chum Salmon. Coho Salmon tend to be at a higher trophic position than Chum Salmon and typically feed on a mixture of zooplankton and fish, compared with zooplankton and jellyfish for Chum Salmon^(14)^.

In 2013 tissue samples were collected from three species of groundfish. Eleven Pacific spiny dogfish (*Squalus suckleyi*) samples were collected in Hecate Strait between 31st May and 14th June, at depths of 95–169 m. Pacific Spiny dogfish is a small shark that inhabits temperate waters off the west coasts of North America. They are a long-lived species with a maximum age in the Pacific population of between 80 and 90 y and a maximum size of 130 cm. They are ovoviviparous with a gestation period of 2 y. Females produce 2–16 pups, on average, between 26 and 27 cm in length at birth. Juvenile dogfish pass through a pelagic phase before taking up bottom residence. Spiny dogfish are capable of impressive migration distances. An extensive tagging project by the Department of Fisheries and Oceans undertaken from 1978 to 1988 found that spiny dogfish tagged off the west coast of Canada migrate as far as the coasts of Japan and Mexico^(15)^.

Ten Pacific Halibut (*Hippoglossus stenolepis*) samples were collected, also in Hecate Strait, between 12th and 20th June at depths of 39–95 m. Pacific Halibut are the largest member of the flatfish family Pleuronectidae; they are found on or near the continental shelf through much of the North Pacific ranging from California to the Bearing Sea and south again to the Sea of Japan. Pacific Halibut are generally found at depths between 10 and 300 m but have been found as deep as 1200 m. Pacific Halibut are long lived with a maximum reported age of 55 y, a maximum length of 267 cm and a maximum weight of 363 kg. Most spawning is believed to occur in Alaskan waters with an ontogenetic migration southwards along the west coast of North America. Tagging studies have shown this species undertakes extensive east and westward migrations along the west coast of North America, as such the entire eastern Pacific Population is considered to be a single stock.

Seven Sablefish (*Anoplopoma fimbria*) samples were collected in Queen Charlotte Sound between 11th and 21st July, at depths of 302–368 m. An additional 20 were collected between 9th and 15th November in Portland Inlet, Chatham Sound, Gil Island, Milbanke Sound and Fitzhugh Sound. Sablefish are found along the eastern north Pacific coast from Baja Mexico to Alaska, along the Aleutian Island chain, and the continental slope in the Bering Sea. In the western north Pacific, they occur from Siberia in the Bering Sea to the Commander Islands in Japan. Adult sablefish are found near bottom over soft substrate, living at depths of up to 2700 m; juveniles migrate inshore for several years, where they can be found in shallow waters, and then migrate offshore as adults. Sablefish are long lived with a maximum reported age of 95 y, a maximum length of 118 cm and weight of 25 kg. Tagging studies carried out by Canadian and US agencies since the late 1970s show that Sablefish undertake extensive movements, including onshore-offshore, along shelf, shelf to seamount and basin-scale movements from British Columbia waters to the Bearing Sea and Baja California.

To address radiological concerns about fish consumption, only the edible portion of a fish is of interest. Therefore, skin and bone were first separated from the flesh before a homogenised sample was prepared for radioactivity measurement.

Radioactive caesium (^134^Cs and ^137^Cs) has been released to the environment due to the Fukushima accident. Both ^134^Cs and ^137^Cs emit beta and gamma radiation^(16)^. ^134^Cs has a radioactive half-life of 2.06 y, yielding one beta particle per transformation with a mean energy of 0.157 MeV. It also emits an average of 2.23 gamma rays per transformation with a mean energy of 0.698 MeV. ^137^Cs has a half-life of 30.17 y, yielding one beta particle per transformation with a mean energy of 0.188 MeV. It decays to ^137^Ba by emitting a gamma ray of energy of 0.662 MeV in 89.8 % of transformations.

Homogenised samples were fully packed in PVC vials (60 mm in diameter and 55 mm in height), and then scanned for gamma-emitting radionuclides by counting on a Gamma Analyst Integrated Gamma Spectrometer (GAM-AN2) coupled with a BEGe detector. The detector was designed for broad energy coverage (3 keV–3 MeV), with enhanced detection efficiency <1 MeV. The gamma spectra were collected by placing the fish sample directly on top of the BEGe detector and counting for several hours. Selected samples were counted up to 24 h in order to achieve a MDC of <1 Bq kg^−1^. When the results of the 24-h counting did not show any activity concentration of caesium, most other samples were counted for 2 h.

To put radiation doses from radioactive caesium intake into perspective, more recent samples were measured for radioactive polonium (^210^Po) commonly found in fish^(17)^. ^210^Po is a naturally occurring radionuclide, a decay product of ^210^Pb in the ^238^U decay chain of the uranium series. It has a half-life of 138.38 d^(14)^. ^210^Po decays to ^206^Pb by emitting alpha particles with energies of 5.3044 MeV (100 %) and 4.5166 MeV (0.00122 %)^(16)^. For each sample harvested in November 2013, 20 g of wet sample was acid digested in a microwave digestion system prior to counting by alpha spectrometry for ^210^Po, with ^209^Po added as a tracer to determine the chemical recovery for the method. Results of radioactivity concentrations are given in the units of Bqkg^−1^ of wet sample weight.

## RESULTS AND DISCUSSION

Results of radioactivity measurements are summarised in Table 1. To date, radioactive caesium in all fish samples measured was below the MDC. The MDCs averaged over all samples were 1.9 Bq kg^−1^ for ^134^Cs and 1.8 Bq kg^−1^ for ^137^Cs. It is worth mentioning that the intervention level for ^137^Cs in food following a nuclear emergency is 1000 Bq kg^−1(18)^.

**Table 1.** Results of radioactivity measurements of fish samples from Canadian west coast.

Fish	Harvested	^134^Cs activity (Bq kg^−1^)	^134^Cs MDC (Bq kg^−1^)	^137^Cs activity (Bq kg^−1^)	^137^Cs MDC (Bq kg^−1^)	^210^Po activity (Bq kg^−1^)	^210^Po MDC (Bq kg^−1^)
Chum	November 2013	<MDC	0.6	<MDC	0.6	1.2	0.3
Chum	November 2013	<MDC	2.1	<MDC	2.2	0.5	0.2
Chum	November2013	<MDC	2.2	<MDC	2.2	1.2	0.3
Chum	November2013	<MDC	2.1	<MDC	2.2	1.0	0.3
Chum	November 2013	<MDC	2.2	<MDC	2.2	1.2	0.3
Chum	November 2013	<MDC	2.2	<MDC	2.2	0.7	0.3
Chum	November2013	<MDC	0.6	<MDC	0.6	0.7	0.2
Chum	November 2013	<MDC	2.0	<MDC	2.0	0.8	0.2
Chum	November 2013	<MDC	2.0	<MDC	2.0	0.6	0.2
Chum	November 2013	<MDC	2.2	<MDC	2.1	1.4	0.3
Chum	November 2013	<MDC	2.3	<MDC	2.3	<MDC	0.3
Chum	November 2013	<MDC	2.2	<MDC	2.1	1.2	0.3
Coho	November 2013	<MDC	2.2	<MDC	2.1	0.3	0.2
Coho	November 2013	<MDC	0.6	<MDC	0.6	<MDC	0.2
Halibut	June 2013	<MDC	2.3	<MDC	2.2	x	
Halibut	June 2013	<MDC	7.3	<MDC	6.2	x	
Halibut	June 2013	<MDC	2.7	<MDC	2.6	x	
Halibut	June 2013	<MDC	6.1	<MDC	5.5	x	
Halibut	June 2013	<MDC	3.3	<MDC	3.1	x	
Halibut	June 2013	<MDC	2.7	<MDC	2.4	x	
Halibut	June 2013	<MDC	2.8	<MDC	2.5	x	
Halibut	June 2013	<MDC	2.6	<MDC	2.2	x	
Halibut	June 2013	<MDC	2.5	<MDC	2.3	x	
Halibut	June 2013	<MDC	2.3	<MDC	2.1	x	
Sablefish	July 2013	<MDC	2.3	<MDC	2.1	x	
Sablefish	July 2013	<MDC	2.2	<MDC	2.1	x	
Sablefish	July 2013	<MDC	2.2	<MDC	2.1	x	
Sablefish	July 2013	<MDC	2.5	<MDC	2.3	x	
Sablefish	July 2013	<MDC	2.7	<MDC	2.4	x	
Sablefish	July 2013	<MDC	2.6	<MDC	2.2	x	
Sablefish	July 2013	<MDC	2.4	<MDC	2.2	x	
Sablefish	November 2013	<MDC	0.3	<MDC	0.2	0.6	0.2
Sablefish	November 2013	<MDC	0.2	<MDC	0.2	0.2	0.2
Sablefish	November2013	<MDC	0.2	<MDC	0.2	0.3	0.2
Sablefish	November 2013	<MDC	0.6	<MDC	0.6	<MDC	0.2
Sablefish	November 2013	<MDC	0.5	<MDC	0.5	0.3	0.2
Sablefish	November 2013	<MDC	0.3	<MDC	0.4	0.4	0.2
Sablefish	November 2013	<MDC	0.6	<MDC	0.4	3.5	0.2
Sablefish	November 2013	<MDC	0.5	<MDC	0.5	0.4	0.2
Sablefish	November 2013	<MDC	0.4	<MDC	0.6	<MDC	0.2
Sablefish	November 2013	<MDC	0.9	<MDC	1.0	0.2	0.2
Sablefish	November 2013	<MDC	0.7	<MDC	1.0	0.4	0.2
Sablefish	November 2013	<MDC	0.6	<MDC	0.4	0.6	0.2
Sablefish	November 2013	<MDC	0.4	<MDC	0.4	0.6	0.6
Sablefish	November 2013	<MDC	0.5	<MDC	1.0	0.9	0.2
Sablefish	November 2013	<MDC	0.7	<MDC	0.3	0.3	0.2
Sablefish	November 2013	<MDC	0.4	<MDC	0.4	0.4	0.2
Sablefish	November 2013	<MDC	1.0	<MDC	0.8	<MDC	0.7
Sablefish	November 2013	<MDC	0.4	<MDC	0.4	0.2	0.2
Sablefish	November2013	<MDC	0.3	<MDC	0.4	<MDC	0.2
Sablefish	November2013	<MDC	0.3	<MDC	0.3	0.3	0.2
Spiny dogfish	June 2013	<MDC	2.7	<MDC	2.5	x	
Spiny dogfish	June 2013	<MDC	2.5	<MDC	2.2	x	
Spiny dogfish	June 2013	<MDC	3.0	<MDC	2.5	x	
Spiny dogfish	June 2013	<MDC	5.2	<MDC	4.7	x	
Spiny dogfish	June 2013	<MDC	4.9	<MDC	4.1	x	
Spiny dogfish	June 2013	<MDC	2.4	<MDC	2.1	x	
Spiny dogfish	June 2013	<MDC	2.6	<MDC	2.2	x	
Spiny dogfish	June 2013	<MDC	2.5	<MDC	2.3	x	
Spiny dogfish	June 2013	<MDC	2.6	<MDC	2.3	x	
Spiny dogfish	June 2013	<MDC	2.3	<MDC	2.2	x	
Spiny dogfish	June 2013	<MDC	2.5	<MDC	0.8	x	
Average		<1.9	1.9	<1.8	1.8	0.63	

x: alpha spectrometry analysis not performed.

In comparison, a 1978 fish survey in various Canadian lakes measured radioactivity in a total of 422 fish samples^(19)^. The ^137^Cs concentration, attributable to historical nuclear weapons testing, varied from non-detectable to 135 Bq kg^−1^. Averaged over all 422 samples, the mean concentration of ^137^Cs was 15 Bq kg^−1^, which is significantly higher than the detection limits applied in this study for ^137^Cs.

In view of historical contamination from nuclear weapons testing, ^137^Cs could still be present in fish harvested in Canada if a much lower detection limit can be achieved for gamma spectroscopic analysis. Although ^137^Cs concentrations in fish could be well below the lowest MDC shown in Table 1, i.e. <0.2 Bq kg^−1^, one could assume in the worst-case scenario that all fish samples would contain ^137^Cs exactly at the detection limit level (i.e. at the MDC). Due to the short half-life of ^134^Cs, there is no reason to assume the presence of historical ^134^Cs in fish harvested in Canada, although trace amounts from the Fukushima-Daiichi accident might be present based on the ocean water sampling data^(2, 7)^. Considering also the observation that ^134^Cs concentrations in fish products from Fukushima and adjacent prefectures in Japan were less than half of ^137^Cs concentrations^(12)^, it is therefore assumed in the worst-case scenario that all fish samples would contain ^134^Cs at exactly half of the detection limit level, i.e. MDA/2.

Naturally occurring ^210^Po was measured in most samples, but significantly lower than the average concentration of 30 Bq kg^−1^ for generic marine fish reported in the literature^(20)^. Since ^210^Po are always present in fish in varying concentrations, a more practical assumption was applied to fish samples with undetectable ^210^Po, i.e. samples with undetectable ^210^Po having, on average, a ^210^Po concentration at half of the detection limit, i.e. MDC/2. The measured ^210^Po activity concentration averaged over 34 fish samples harvested in November 2013 was 0.63 Bq kg^−1^.

Radiation doses are calculated for one fish meal of 150 g and several fish meals up to 1 kg. Results are summarised in Table 2. They are estimated doses based on the measurements and assumptions described above, for children (5-y old) and adults, respectively. Even in this assumed worst-case of ^134^Cs and ^137^Cs contamination, the resulting radiation doses would still be much less than radiation doses from naturally occurring radionuclides commonly found in fish. Doses from naturally occurring ^210^Po are ∼92 times higher in children and 18 times higher in adults than the ^137^Cs and ^134^Cs combined doses.

**Table 2.** The assumed worst-case radiation doses for children (5 y old) and adults due to intake of one fish meal (150 g) and several fish meals up to 1 kg.

	Assumed concentration (Bq kg^−1^)	DC (nSv Bq^−1^)		µSv (from 150 g)		µSv (from 1 kg)	
		Children	Adults	Children	Adults	Children	Adults
^134^Cs	0.95	13	19	0.0019	0.0027	0.012	0.018
^137^Cs	1.8	9.6	13	0.0026	0.0035	0.017	0.023
^210^Po	0.6	4400	1200	0.42	0.11	2.77	0.76

For an adult who consumes 50 kg of fish per year, the assumed worst-case dose from potential exposure to ^134^Cs and ^137^Cs will be 2 µSv, which is <1/1000 of the annual natural background radiation dose in Canada^(21)^, and of no radiological health concern. The ^210^Po dose from consuming 50 kg of similar fishes studied here would be 38 µSv. Even though much higher than 2 µSv from assumed ^134^Cs and ^137^Cs contamination, the resulting ^210^Po dose is still a very small fraction of the annual dose from exposure to natural background radiation. Therefore, fish, such as salmon and groundfish, from Canada's west coast are of no real health concern for both radiation contaminants and naturally occurring radionuclides. Similar conclusions were obtained for marine biota collected off Japan and California^(17, 22)^.

## CONCLUSIONS

None of the fish samples analysed in this study contained any detectable levels of ^134^Cs and ^137^Cs under given experimental setting with a detection limit of ∼2 Bq kg^−1^. Fish (such as salmon and groundfish) from the Canadian west coast are of no health concern for both radiation contaminants and naturally occurring radionuclides.

As simulations predicted, in the near future, the radioactive water plume could reach the areas where these fish are rearing^(1)^. Even in this case, it is expected that levels of radioactive contaminants in fish will remain well below Health Canada guidelines for food and likely still below the detection limit of a few Bq kg^−1^. Nonetheless, further monitoring of ^134^Cs and ^137^Cs, especially the long-lived ^137^Cs, in ocean water and seafood will help confirm these assessments and ensure public safety.
